# Aromatic Tricyanoethylenes a New Class of ‘Compact’ Photoinitiators for One- and Two-Photon Photopolymerization

**DOI:** 10.3390/polym18080958

**Published:** 2026-04-14

**Authors:** Elnara R. Zhiganshina, Tatyana S. Lyubova, Anastasia E. Tarakanova, Maxim V. Arsenyev, Roman S. Kovylin, Natalia D. Anisimova, Yuri V. Polushtaytsev, Konstantin A. Kozhanov, Anastasia V. Pisarenko, Diana Ya. Aleynik, Marfa N. Egorikhina, Alexei Vitukhnovsky, Larisa G. Klapshina, Sergey A. Chesnokov

**Affiliations:** 1 G.A. Razuvaev Institute of Organometallic Chemistry, Russian Academy of Science (RAS), Tropinin St., 49, 603137 Nizhny Novgorod, Russia; zhiganshinae@mail.ru (E.R.Z.); anasta5ia.tar@yandex.ru (A.E.T.); mulnir@yandex.ru (R.S.K.);; 2 Moscow Institute of Physics and Technology, National Research University, Institutskii Per. 9, 141700 Dolgoprudny, Russia; 3 Federal State Budgetary Educational Institution of Higher Education, Privolzhsky Research Medical University of the Ministry of Health Care of the Russian Federation, Minin and Pozharsky Sq. 10/1, 603950 Nizhny Novgorod, Russia; daleynik@yandex.ru (D.Y.A.);; 4 Lebedev Physical Institute, Russian Academy of Sciences, Leninskii Prospect 53, 119991 Moscow, Russia

**Keywords:** aryltricyanoethylenes, photoinitiating system, two-photon photopolymerization, cytotoxicity

## Abstract

In this paper, we consider a series of new compact A-π-D photoinitiators consisting of donor aromatic fragments (naphthalene, anthracene, phenanthrene, pyrene and perylene) and a strong acceptor tricyanoethylene group—aryltricyanoethylenes (ArTCNEs). Spectral, photophysical, and electrochemical characteristics of ArTCNEs are studied. One-photon (with LED@405 nm) and two-photon (λ = 780 nm, impulse duration of 100 fs) photopolymerization of PETA can be effectively initiated by ArTCNEs with the tertiary amine *N,N*-dimethylcyclohexylamine DMCHA and/or the iodonium salt diphenyliodonium chloride Iod. Based on results of experiments on photodegradation, photopolymerization and EPR spectroscopy, a photoinitiation mechanism of radical photopolymerization was proposed for two-component (AntTCNE/DMCHA) and three-component (AntTCNE/DMCHA/Iod) initiating systems. The composition containing PerTCNE/DMCHA as a photoinitiator demonstrated the best reactivity under two-photon nanolithography conditions: the polymerization threshold was 2 mW at a laser beam scanning speed of 100 μm/s, and the widest fabrication window of 11 mW was typical for it. As an example, 3D “cage” structures were fabricated using the AntTCNE-based composition, and the test structure resolution parameters, such as the minimum line width and the distance between lines of 80 and 400 nm, respectively, were achieved. MTT experiments with human dermal fibroblasts showed promising preliminary biocompatibility of the resulting polymers, which opens up possibilities for using the obtained materials in biological applications.

## 1. Introduction

**Scheme 1:**
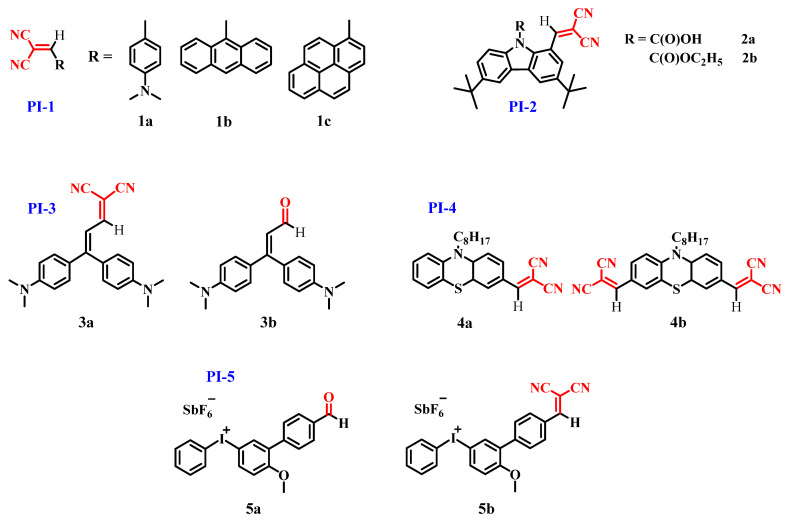
A-π-D type photoinitiators based on malononitrile.

In recent years, photopolymerization has become the most preferred method for creating materials with many applications in the fields of coatings [1,2,3], tissue engineering [4,5,6], photolithography [7,8,9], manufacturing of microfluidic devices [10,11,12], 3D printing [13,14,15], etc. Compared to other polymerization methods, this process can take place in a short period of time (a few minutes or seconds) at room temperature without the use of solvents. In addition, photoprocesses can be localized and time-controlled, which is an undeniable advantage of using this method in one- and two-photon stereolithography for creating macro and micro 3D objects of a given geometry (for example, optical waveguides [16,17], photonic crystals [18,19], drug delivery systems [20,21], functional microdevices based on polymers [22,23], biomaterials [24,25], metamaterials [26,27], etc.). First of all, 3D printing accuracy depends on the mixture of the photoresist or photopolymerizable composition (PPC) and, in particular, on the nature of the photoinitiator or photoinitiating system. In this regard, researchers have developed a variety of photoinitiators for both one- and two-photon photopolymerization, among which push–pull dyes hold a leading position. They represent compounds containing donor and acceptor fragments linked to each other by a π-bridge. The ability to regulate the absorption range by varying the strength of electron-donating and electron-accepting characteristics makes push–pull dyes convenient objects of research as photoinitiators of free-radical and cationic polymerization [28,29,30], and also as photosensitizers in nonlinear optics [31,32], organic photovoltaic systems [33,34], organic field-effect transistors [35], organic light-emitting diodes [36], etc., due to the presence of efficient intramolecular charge transfer from donor to acceptor and a low-energy band gap between the HOMO and LUMO orbitals. In the literature, the acceptor structure is often represented by a carbonyl group and is rarely modified; there, the donor fragment or the π-conjugated system is mainly subject to change. Altering the structure of the acceptor fragment and thus adjusting the properties of dyes is possible by switching from a carbonyl group to a dicyanoethylene group through a simple Quenenagel condensation reaction [37]. Many types of A-π-D dyes have been studied in this field [38,39,40], but only a few of them have been used as photoinitiators of one- and especially two-photon polymerization (Scheme 1) [41,42,43,44,45]. In [41], the influence of the nature of the donor fragment of dicyanoethylene dyes (PI-1, Scheme 1) on the photoinitiation ability in the ring-opening polymerization of epoxy resin was studied. It was found that a three-component system consisting of **1a**, amine (*N*-vinylcarbazole NVC), and iodonium salt had the highest initiation efficiency (conversion reached 50% in 15 min when the system was irradiated with a 457 nm laser diode). However, under the same conditions the systems based on **1b** and **1c** did not initiate polymerization (reachable conversion <5%). The presence of a carboxyl group in the carbazole fragment of push–pull dyes (PI-2, **2a**, Scheme 1) leads to an increase in the rate of photolysis (LED@455 nm) and photopolymerization due to the easier protonation reaction of the carboxylic acid compared to the ester group **2b** [42]. When comparing the photoinitiating capacity of Michler ketone derivatives **3a** and **3b** (PI-3, Scheme 1), which differ in the nature of the acceptor fragments, the absorption band of these compounds should be taken into account [43]. The introduction of a malononitrile group shifts the absorption spectrum maxima to the long-wavelength region by 120 nm. Therefore, exposing PPCs containing the above dyes in a composition with NVC and an iodonium salt to 532 nm light causes cationic polymerization of the epoxy resin in the presence of **3a** with a monomer conversion of 20% in 12 min, whereas **3b** does not initiate polymerization. However, illuminating the same systems with light of 405 nm wavelength results in the formation of a polymer with a conversion of ~40–50%. The reactivity of asymmetric A-π-D and symmetric D-π-A-π-D structures of dicyanoethylene photoinitiators in the photopolymerization of trimethylpropane triacrylate TMPTA was compared using phenothiazine dyes **4a** and **4b** (PI-4, Scheme 1) as an example [44]. The photoinitiating activity of **4b** in the polymerization of TMPTA in two- and three-component systems is comparable. However, there is a conversion jump for **4a**, and the authors attribute it to the effective photocatalytic properties of this compound, which appear upon the addition of an amine. The authors of [45] proposed an interesting design of photoinitiating systems, which are charge transfer complexes (CTCs) based on iodonium salts (PI-5, Scheme 1) and tertiary amines. Compared to iodonium salts with a carbonyl group **5a**, dicyanoethylene group **5b** in the iodonium salt structure promotes more efficient charge transfer between molecules, which in turn facilitates the formation of CTCs. It was found that these compounds initiated both one-photon and two-photon photopolymerization of oligoesters(meth)acrylates. Also, in the Iod2 composition, the lines in the “grid” structures were narrowed from 50 µm to 16 µm, and the relief became clearer after reducing the IR laser power from 35 mW to 30 mW.

In general, such systems are classified as type II photoinitiators, meaning that a co-initiator is required to generate initiating radicals, which is often the dye molecule itself’s push–pull structure, as well as amines, thiols, and alcohols. Furthermore, onium salts (sulfonium or iodonium salts) are regularly used as additives to PPCs containing type II photoinitiators and/or a hydrogen donor. When studying photopolymerization, two- and/or three-component systems are usually used, including iodonium salts (Iod) and amines of various structures.

**Scheme 2:**
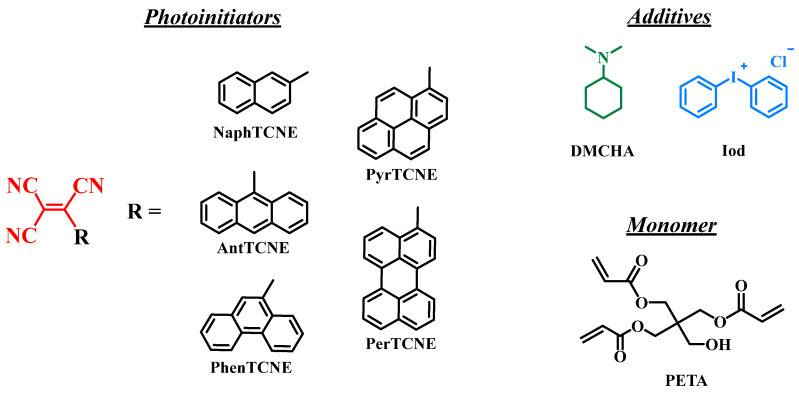
Chemical structures of the photoinitiators, additives, and monomers used in this study.

The objects of research in this work are aryltricyanoethylenes (ArTCNEs). They include condensed aromatic substituents—naphthalene, anthracene, phenanthrene, pyrene and perylene (D)—conjugated with a tricyanoethylene fragment, which is highly electron-deficient (A). These compounds have already proven themselves as building blocks for the synthesis of cyanoporphyrazine macrocycles [46], which have a range of sensory and therapeutic properties. This molecular structure and the compactness of the molecules make ArTCNEs promising objects as a new class of two-photon photoinitiators, while most of such initiators are structures possessing an extended π-electron system with donor and acceptor fragments (D-π-A-π-D, A-π-D-π-A, etc.). It should be noted that the characteristics of two-photon absorption for perylene tricyanoethylene were studied by Z-scan method and the value of the two-photon absorption cross-section was determined to be 1.85 GM [46], which is close to the value for Michler’s ketone (~2 GM). The last one is used as an initiator of two-photon polymerization despite the low values of the two-photon absorption cross-section [47]. We have previously found that the transition from the anthracene-containing dicyanoethylene known in the literature to the tricyanoethylene derivative leads to a shift in the reduction potential by 0.47 V to the anodic region, a bathochromic shift in the long-wave absorption band from λ_max_ = 427 nm to λ_max_ = 525 nm in acetonitrile and, most importantly, an increase in the efficiency of the initiation of one-photon photopolymerization of the dimethacrylate oligocarbonate OCM-2 (LED@405 nm) by more than five times [48]. Photophysical and electrochemical characteristics of ArTCNEs, including photoconversion of dyes in the presence of an amine and/or iodonium salt, were studied. In addition, the photoinitiating activity of these compounds was explored under conditions of one- and two-photon polymerization of PETA. The final polymers were evaluated for cytotoxicity using a test culture of human dermal fibroblasts. The structures of the compounds used in this study and their designations are shown in Scheme 2.

## 2. Materials and Methods

### 2.1. Materials

Diphenyliodonium chloride **Iod** (99%, ‘Aldrich’, St. Louis, MO, USA), *N,N*-dimethylcyclohexylamine **DMCHA** (99%, ‘Aldrich’, St. Louis, MO, USA), *N*-tert-butyl-*α*-phenylnitrone **PBN** (99%, ‘Aldrich’, St. Louis, MO, USA), and pentaerythritol triacrylate **PETA** (99%, ‘Aldrich’, St. Louis, MO, USA) were used without purification. Compounds NaphTCNE, AntTCNE, PhenTCNE, PyrTCNE, and PerTCNE were synthesized according to the methods described in [46,49,50].

### 2.2. UV–Visible Spectroscopy and Photolysis Experiments

Electronic absorption spectra were measured on an SF-56 spectrometer (LOMO, Saint-Petersburg, Russia), and luminescence spectra were recorded using a Perkin Elmer LS 55 (PerkinElmer, Shelton, CT, USA) at room temperature in a 1 cm thick quartz cuvette. For steady-state photolysis experiments, ArTCNEs were dissolved in acetonitrile, DMCHA and/or Iod were added, and then they were irradiated with LED@395 nm (30 mW) at predetermined time intervals. All experiments were carried out in air.

### 2.3. Electrochemistry

The redox potentials were determined using the cyclic voltammetry method (CV) in a three-electrode cell (potentiostat–galvanostat “PS-50” (SmartStat, Boston, MA, USA)) under an argon atmosphere. The working electrode was the stationary glassy-carbon electrode (d = 2 mm); the auxiliary electrode was the platinum wire. The reference electrode was Ag/AgCl/KCl (sat.) with a waterproof diaphragm. The potential scan rate was 0.2 V/s. The solvent was acetonitrile. The concentration of ArTCNE was 0.005 M. The background electrolyte was 0.1 M (NBu_4_)ClO_4_ (‘Aldrich’), twice recrystallized from an aqueous ethanol solution and dried in vacuum (48 h) at 50 °C.

### 2.4. Electron Spin Resonance Spin-Trapping Experiment

EPR spectra were recorded by a Bruker-Magnettech MS5000 (X-band) spectrometer (Bruker BioSpin, Berlin, Germany) using evacuated 3 mm standard ampoules. The samples were exposed to a 3 W 395 nm LED lamp in the spectrometer’s cavity. The parameters of the spectra were obtained by simulation with EasySpin 6.0.6 software [51].

### 2.5. Real-Time Photopolymerization Experiments

The kinetics of one-photon photopolymerization of PETA were studied by FTIR spectroscopy. PPC was prepared by dissolving ArTCNEs (0.018 M), amine (0.18 M) and/or iodonium salt (0.018 M) in PETA. The spectra were recorded using an FT-801 spectrometer (Simex, Novosibirsk, Russia) with the TR attachment with diamond crystal. An integrated LED system (λ = 405 nm) with adjustable illumination power on the PPC surface 0–48 mW/cm^2^ was used for photopolymerization. The PETA conversion was calculated from the change in the intensity of the absorption band of acrylate groups at 806 cm^−1^ relative to the unchanged intensity of the band corresponding to vibrations of the C=O group at 1720 cm^−1^. Each curve of polymerization was an average result of three experiments differing in maximum superficial polymerization rate and limiting conversion by no more than 5%.

### 2.6. Two-Photon Induced Photopolymerization

The PPCs were the same as for the one-photon polymerization experiment. The two-photon photopolymerization study was provided with Nanoscribe Photonic Professional (Nanoscrbe, Gmbh, Eggenstein-Leopoldshafen, Germany) for Direct Laser Writing lithography (DLW). The PPC was exposed with a femtosecond focused laser beam (λ = 780 nm). The laser radiation was focused with a high-aperture (NA = 1.4) Zeiss PlanApo objective (Carl Zeiss AG, Baden-Württemberg, Germany). Precise control of laser radiation power, positioner movement speed, and trajectory were provided by Nanoscribe Photonic Professional hardware and software.

### 2.7. Scanning Electron Microscopy

Images of polymer microstructures obtained on a glass substrate by DLW nanolithography were recorded using a Regulus SU8100 ultra-high-resolution scanning electron microscope (Hitachi, Hitachinaka, Japan). The samples were examined with a conductive platinum coating at an accelerating voltage of 0.7 to 1.0 kV.

### 2.8. Preparation of Polymer Samples of polyPETA for Cytotoxicity Studies

PPC was prepared by dissolving ArTCNEs (0.018 M), amine (0.18 M), and/or iodonium salt (0.018 M) in PETA. A mold cavity between two silicate glasses separated by 1.5 mm thick damping gasket was filled with the composition and illuminated with LED@395 nm for 1 h. The sample was then removed from the mold. The resulting polymer was ground in a mortar to a fine powder and then extracted with hot isopropyl alcohol in a Soxhlet apparatus (C. Gerhardt GmbH & Co, Koenigswinden, Germany) for 20 h.

### 2.9. Cytotoxicity Test

The cytotoxicity of samples based on ArTCNEs was assessed using test cultures of human dermal fibroblasts obtained in the Biotechnology Laboratory of the Federal State Budgetary Educational Institution of Higher Education Privolzhsky Research Medical University of the Ministry of Health Care of the Russian Federation. The level (rank) of cytotoxicity was determined using the standard MTT test [52]. Each polymer sample was filled with DMEM/F12 culture medium containing 1% antibiotics (penicillin–streptomycin) and 2% fetal bovine serum. The volume of the medium was determined according to the sample weight from a ratio of 100 mg: 1 mL and placed in a CO_2_ incubator for extraction for 24 h (daily extraction) under standard conditions (37 °C, 5% CO_2_, high humidity). After that, the obtained extracts containing a large amount of fine powder were precipitated by centrifugation and then sterilized by filtration successively through 0.8 μm and 0.22 μm filters. During the process of obtaining extracts and their dilutions, the pH remained stable at 7.2–7.4. The prepared extracts were diluted with growth medium in the following ratioa: 1:1; 1:2; 1:4; and 1:8. The extracts and their dilutions were then pipetted onto a test culture, which had been pre-cultured for 24 h, in a flat-bottomed 96-well plate (Costar, USA), into 8 wells of each plate. The growth medium DMEM/F12 with the addition of antibiotics (penicillin/streptomycin), glutamine and 10% fetal bovine serum (FBS) was used for cultivation. All reagents used, unless otherwise stated, were from PanEco (Moscow, Russia). The control cells were those from the experimental culture, in which the nutrient medium was replaced with a standard nutrient medium after 24 h. After 72 h of cultivation with extracts, the state of the culture on the surface of the experimental and control wells was assessed using a Leica DMI 3000 B inverted microscope (LeicaMicrosystems, Wetzlar, Germany, LAS v. 4.3 software, LeicaMicrosystems, Wetzlar, Germany) and recorded in a photo archive. After photofixation, 20 μL of MTT (Methylthiazolyldiphenyl-tetrazolium bromide, neoFroxx GmbH, Einhausen, Germany)—(3-(4,5-dimethylthiazol-2-yl)-2,5-diphenyl-tetrazolium) at a concentration of 5 mg/mL was added to each well of the plate and incubated for another 3 h in a CO_2_ incubator under standard conditions. After 3 h, the fluid from the wells was removed and replaced with an equal volume of DMSO solution and the optical density was recorded at 540 nm on an INFINITE F50 analyzer, Tecanphotometer (TecanAustriaGmbH, Grödig, Austria). Then the relative growth intensity was calculated and the level (rank of cytotoxicity) was determined.

The relative growth rate (RGR or *V*, %) was determined as a percentage using the following formula:

(1) $$\mathrm{RGR}\;=\;\frac{\mathrm{mean}\mathrm{OD}\mathrm{of}\mathrm{test}\mathrm{compound}}{\mathrm{mean}\mathrm{OD}\mathrm{of}\mathrm{control}}\times 100$$

where RGR is the relative growth rate and OD is the optical density.

The level (rank) of cytotoxicity was determined according to (DIN EN ISO 10993-5:2009 [53]): RGR ≥ 70% is no cytotoxicity (rank 0–1) and at RGR < 70 the materials are cytotoxic to varying degrees (ranks 2–5).

## 3. Results and Discussions

### 3.1. Light Absorption Properties of ArTCNEs

**Figure 1:**
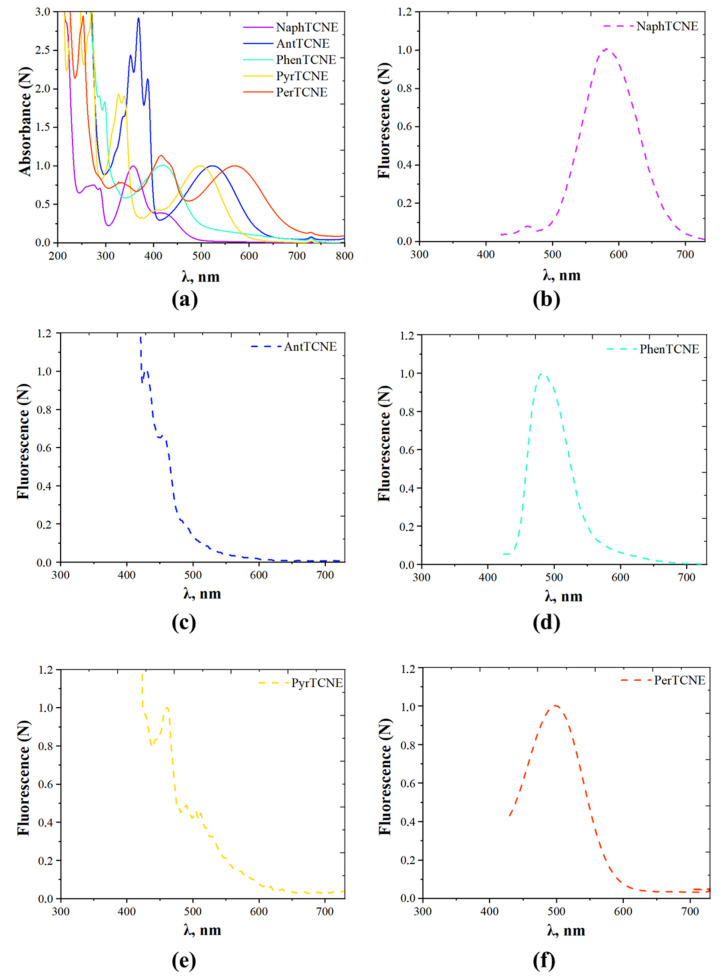
Normalized electronic absorption (**a**) and fluorescence spectra of ArTCNE (**b**–**f**) in acetonitrile. [ArTCNE] = 10^−5^ M. Fluorescence excitation wavelength is 400 nm.

The aryltricyanoethylenes are low-molecular-weight fluorescent dyes. Table 1 shows the absorption band maxima of unsubstituted arylenes and the spectral characteristics of the corresponding ArTCNEs. It is evident that the introduction of the electron-withdrawing tricyanethylene (TCNE) group into the structure of arylenes leads to the appearance of a new band in the absorption spectra in the longer-wavelength region of the spectrum. Previously, this band for AntTCNE, PyrTCNE and PerTCNE was assigned by the authors [46] to the charge transfer band, which made it possible to assign the long-wave absorption bands for NaphTCNE and PhenTCNE to the charge transfer band as well. The electronic absorption spectra of ArTCNEs, normalized by the intensity of the charge transfer band, are shown in Figure 1a. The position and shape of the charge transfer band strongly depend on the nature of the aromatic fragment. Thus, for NaphTCNE, the charge transfer band has a maximum at λ = 358 nm and a shoulder in the region of 400 nm. For AntTCNE, the charge transfer band has a maximum at λ = 525 nm; for PhenTCNE λ = 425 nm; and for PyrTCNE and PerTCNE λ = 500 and λ = 570 nm, respectively. The data are presented in Table 1. The maximum molar extinction coefficient of the charge transfer band is for NaphTCNE (ε = 8160 M^−1^ cm^−1^); the minimum is for AntTCNE (ε = 1900 M^−1^ cm^−1^). It can be assumed that this difference is associated with greater steric hindrance to the rotation of the TCNE fragment relative to the aryl fragment in the AntTCNE molecule compared to the NaphTCNE molecule. The dihedral angle in the crystal structure of AntTCNE is 65.9°, which is ~1.3 times larger than for PhenTCNE (Figure S1, Supplementary Materials), PyrTCNE and PerTCNE [46]. The structure of NaphTCNE is coplanar, which results in a high extinction coefficient [49].

Two-photon photolithography systems use a titanium–sapphire laser with a wavelength of ~800 nm. Accordingly, the photophysical characteristics of the dyes, the kinetics of photodegradation of the initiating system, and one-photon photopolymerization were studied using radiation sources in the 400 nm region. Exciting at a wavelength of 400 nm causes fluorescence for all aryltricyanethylenes. The normalized fluorescence spectra of ArTCNE are shown in Figure 1b,f; the positions of the emission band maxima are given in Table 1.

### 3.2. Electrochemistry

**Figure 2:**
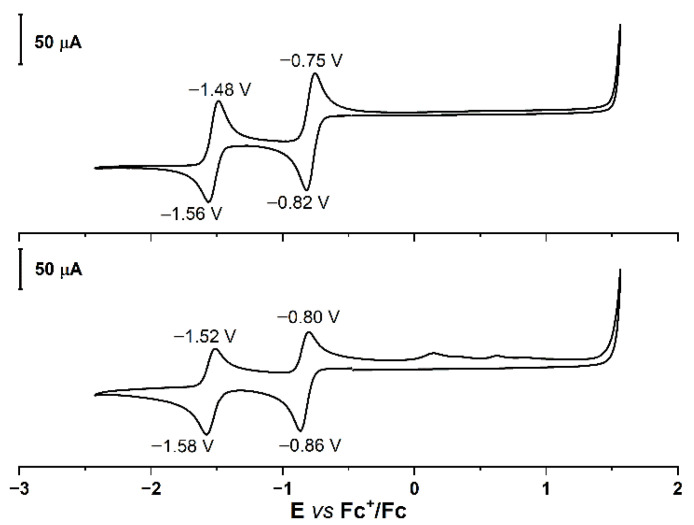
CV curves for NaphTCNE (**a**) and PhenTCNE (**b**). [ArTCNE] = 0.005 M. [NBu_4_ClO_4_] = 0.1 M, argon, scan rate 200 mV/s.

The redox properties of the aryltricyanoethylenes were studied by the cyclic voltammetry method (CV) in acetonitrile compared with Ag/AgCl/KCl (sat.). In a previous study [46], CV data for AntTCNE, PyrTCNE and PerTCNE were already presented. In this section, we present the electrochemical characteristics for NaphTCNE and PhenTCNE (Figure 2). For all compounds of this series, reversible reduction of the tricyanethylene fragment and irreversible oxidation of the aromatic fragment are observed. The values of reduction potentials (E^1^_1/2 Red_) for all aryltricyanoethylenes are observed in the region of –0.82 V, while the values of oxidation potentials (E_Ox_) change significantly. The results of electrochemical studies and the calculated values of HOMO and LUMO energies are given in Table 2. It can be seen that when moving from PhenTCNE to PerTCNE, the value of ΔE decreases significantly. These data correlate with the bathochromic shift in the charge transfer band in the UV spectra of these dyes.

### 3.3. Steady-State Photolysis of ArTCNE

**Figure 3:**
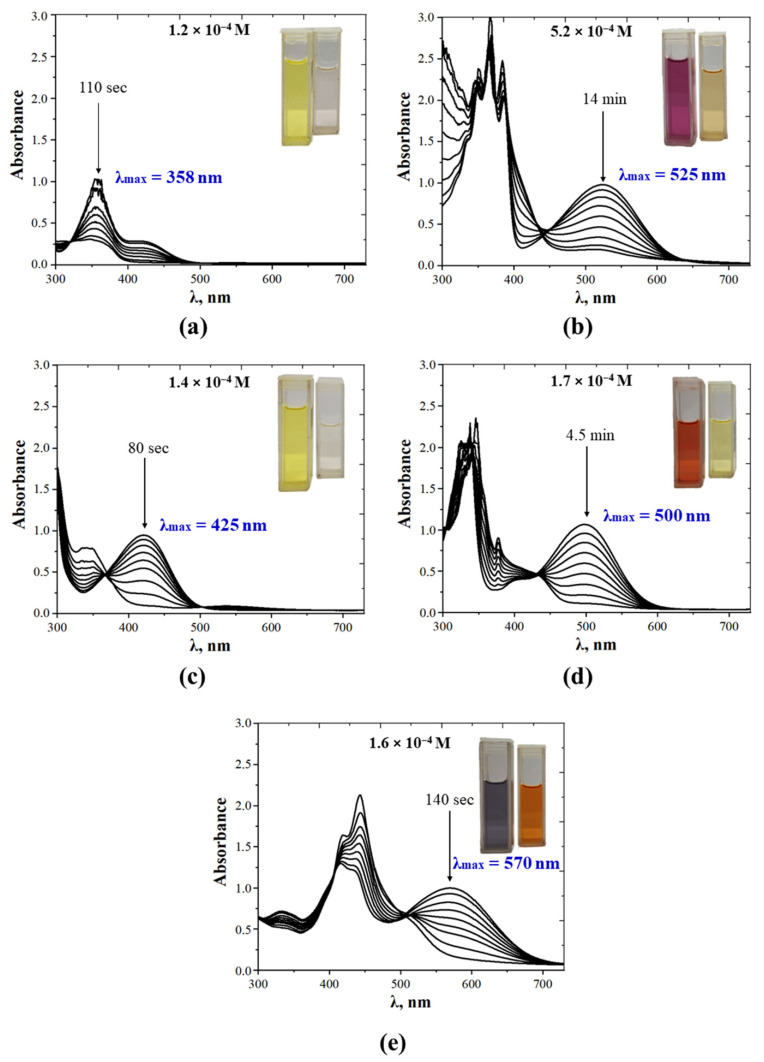
The spectral changes in the NaphTCNE (**a**), AntTCNE (**b**), PhenTCNE (**c**), PyrTCNE (**d**), and PerTCNE (**e**) solutions in acetonitrile in the presence of DMCHA under irradiation with LED@395 nm. ArTCNE:DMCHA = 1:10 mol; P = 33 mW/cm^2^.

Exposing solutions of ArTCNE in acetonitrile in the presence of *N,N*-dimethylcyclohexylamine (DMCHA) and DMCHA with diphenyliodonium chloride (Iod) to the light of LED@395 causes a decrease in the intensity of the charge transfer band in the electronic absorption spectra (Figure 3). Also, Figure 3 shows photographs of ArTCNE solutions with DMCHA in spectrophotometric cuvettes before and after illumination (cuvettes on the left and right, respectively). The solutions with NaphTCNE and PhenTCNE are completely photobleached, while solutions of other ArTCNEs change color. In the absence of amine, irradiation with LED@395 does not cause changes in the absorption spectra of ArTCNE and ArTCNE/Iod solutions.

Analysis of changes in the spectral characteristics of ArTCNE/DMCHA solutions upon irradiation shows that in all cases, there is a decrease in the intensity of the charge transfer band and an increase in the absorption intensity in the shorter-wavelength region of the spectrum. For NaphTCNE, PhenTCNE and PyrTCNE, clear isosbestic points can be observed.

**Figure 4:**
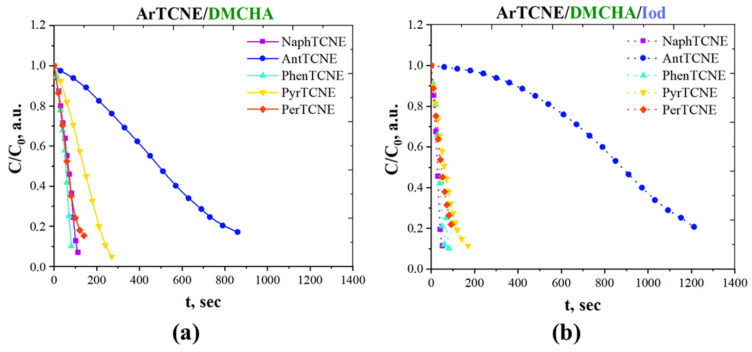
The photodegradation curves of ArTCNE in the presence of DMCHA (**a**) and DMCHA/Iod (**b**) under irradiation with LED@395 nm in acetonitrile. [ArTCNE] = 10^−4^ M; ArTCNE:DMCHA:Iod = 1:10:1 mol; P = 33 mW/cm^2^.

Figure 4 shows the changes in ArTNCE concentration over time in relative units when the process is carried out in the presence of DMCHA (Figure 4a) and DMCHA/Iod (Figure 4b). The C/C_0_ values were determined spectrophotometrically as the ratio of the current optical density at λ_max_ of the charge transfer band to the initial value of the optical density at λ_max_. It can be seen that for NaphTCNE, PhenTCNE and PerTCNE in the presence of DMCHA, the dependencies C/C_0_ = f(t) are close to each other and they are linear. The photoconversion of PyrTCNE occurs at a lower rate, and the dependence C/C_0_ = f(t) is also close to linear. The photoconversion of AntTCNE is the slowest, and the photodegradation curve has an S-shaped character. Adding Iod to the system, on the one hand, accelerates the photodegradation of NaphTCNE, PhenTCNE, PerTCNE and PyrTCNE and eliminates the differences between the C/C_0_ = f(t) dependencies for these ArTCNEs, and on the other hand, further slows down the photoreaction of AntTCNE.

Table 3 shows the values of the initial rates of photodegradation (*V*_0_) and the half-life periods (*τ*_1/2_) at which the optical density of the solution decreases by half for two- and three-component systems under the experimental conditions. The rate of photodegradation of the most reactive NaphTCNE, PhenTCNE, and PerTCNE in the presence of DMCHA is 2.5–3 times higher than that of the pyrene derivative and almost an order of magnitude higher than AntTCNE. Adding Iod to the system increases the photoconversion rate of NaphTCNE, PhenTCNE, PerTCNE and PyrTCNE by 1.5–3 times and eliminates the differences between them. In contrast to this effect, the addition of Iod reduces the rate of photodegradation of AntTCNE by 7 times and the difference in reactivity between AntTCNE and other ArTCNEs increases by an average of two orders of magnitude.

### 3.4. Kinetic Analysis of One-Photon Photopolymerization

**Figure 5:**
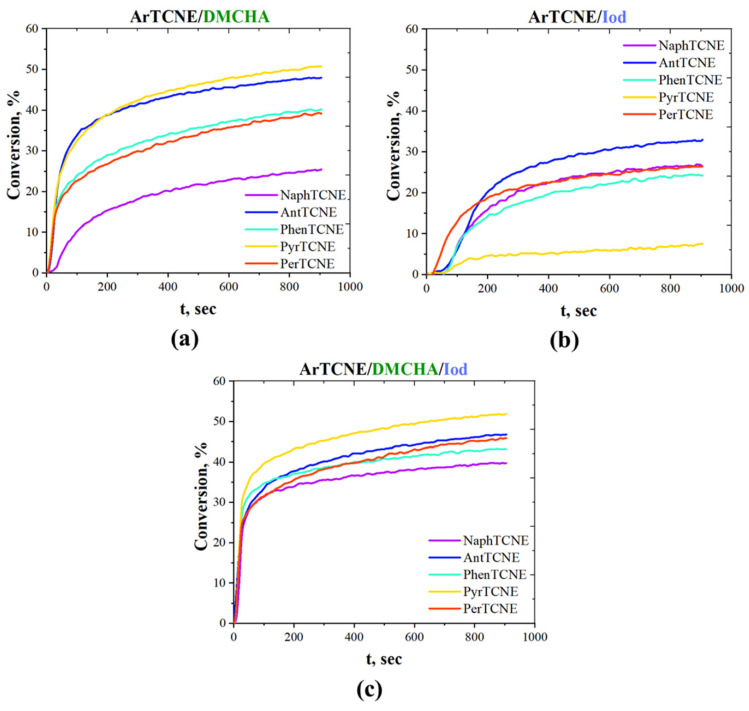
Photopolymerization kinetics of ArTCNE/DMCHA (**a**), ArTCNE/Iod (**b**), and ArTCNE/DMCHA/Iod (**c**) using PETA as monomer under air; the light source is at 405 nm with 33 mW/cm^2^ irradiation intensity. [ArTCNE] = 0.018 M; [Am] = 0.18 M; [Iod] = 0.018 M.

To assess the influence of the nature of ArTCNEs and the addition of amine and/or iodonium salt on the rate of photopolymerization, the kinetics of one-photon photopolymerization of PETA initiated by the ArTCNE/DMCHA, ArTCNE/Iod and ArTCNE/DMCHA/Iod systems was studied. The maximum possible concentration of ArTCNE in PETA was 0.018 M; the molar ratios were ArTCNE:DMCHA = 1:10 mol and ArTCNE:Iod = 1:1 mol (corresponding to an amine concentration of 2 wt.%). The studies were conducted using FTIR spectroscopy; the PPC was irradiated with LED@405 nm. The kinetic curves for PETA photopolymerization in the presence of the abovementioned photoinitiating systems are shown in Figure 5. Table 4 presents the kinetic parameters of photopolymerization. It should be noted that ArTCNE without the addition of DMCHA and Iod does not initiate polymerization of PETA.

Analysis of the kinetic curves shows the following: All ArTCNEs in the presence of DMCHA initiate photopolymerization of PETA (Figure 5a). The most effective are AntTCNE and PyrTCNE (maximum PETA conversion is approximately 50%), followed by PhenTCNE and PerTCNE (maximum PETA conversion is approximately 40%). NaphTCNE is the least effective with a maximum PETA conversion of ~25%. The AntTCNE/DMCHA pair exhibits the highest reactivity as a polymerization photoinitiator: the photopolymerization rate is 0.97 s^−1^ and PETA conversion is 48% (Table 4). The worst performance is demonstrated by the NaphTCNE/DMCHA pair, for which the photopolymerization rate is 0.14 s^−1^ (6.6 times less than for AntTCNE/DMCHA). Essential slowdown in photopolymerization and decrease in the maximum conversion of PETA occur if DMCHA is replaced with Iod, and more significantly in the presence of PyrTCNE, which with Iod almost does not initiate polymerization (Figure 5b). The transition to the three-component photoinitiating system ArTCNE/DMCHA/Iod leads to an improvement in the kinetic characteristics of polymerization of PETA for each of the systems. Moreover, the maximum photopolymerization rate is virtually independent of the nature of ArTCNE; the difference in W_max_ values is less than 2%. In general, the difference in the maximum process rates between ArTCNE/DMCHA and ArTCNE/DMCHA/Iod is in the range of 1.2–8 times, with the minimum difference observed for PPCs with AntTCNE and the largest difference for NaphTCNE. For other dyes, the presence of Iod increases the rate of photopolymerization by approximately 1.5 times.

### 3.5. Electron Spin Resonance Spin-Trapping (ESR-ST) Experiment and Photopolymerization Mechanism

**Figure 6:**
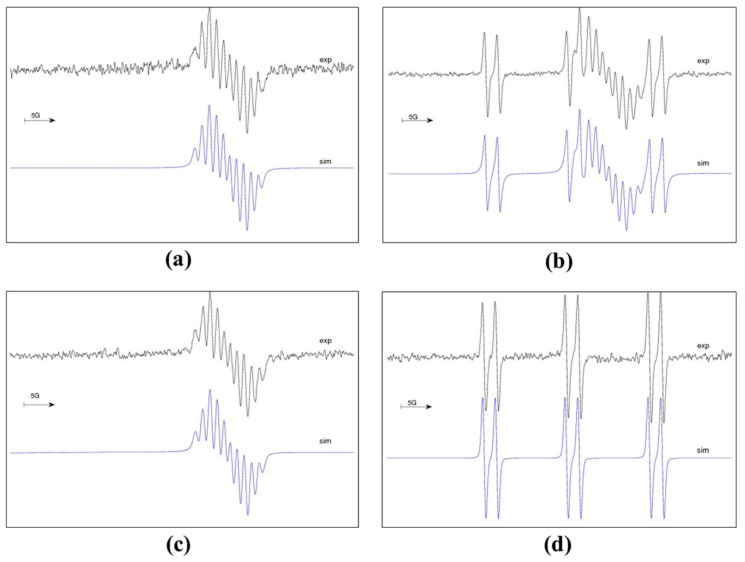
EPR spectra of AntTCNE/DMCHA (**a**), AntTCNE/DMCHA (PBN) at the time of irradiation (**b**), AntTCNE/DMCHA (PBN) after irradiation (**c**), AntTCNE/Iod (PBN) after irradiation (**d**).

To demonstrate the radical nature of the polymerization process, an EPR study was conducted on the reaction mixtures of ArTCNE/DMCHA, ArTCNE/DMCHA/Iod, and ArTCNE/Iod in benzene under irradiation with LED at 395 nm. To determine the structure of short-lived radicals, a spin trap of *N*-tert-butyl-*α*-phenylnitrone (PBN) was used. Experiments were performed for AntTCNE, PyrTCNE, and PhenTCNE. The obtained results were similar for all cases, as shown in Figure 6 for AntTCNE. It was found that irradiation of the AntTCNE/DMCHA mixture in benzene generated a fairly stable radical species containing three nonequivalent nitrogen atoms and a hydrogen atom (the EPR spectrum was satisfactorily simulated with the following parameters: g_i_ = 2.00574 and constants a_N_ = 1.27 G (1N), a_N_ = 1.40 G (1N), a_N_ = 1.11 G (1N), and a_H_ = 3.36 G (1H)) (Figure 6a). The hyperfine splitting constants on ^14^N nuclei most likely relate to the TCNE fragment; the additional splitting on ^1^H is probably due to the abstraction of hydrogen from DMCHA. Irradiation of a similar solution containing a PBN spin trap results in a superposition of signal 1 and the product of the interception of aminoalkyl radicals of PBN (Figure 6b). Initially, a triplet of 1:1:1 doublets (g_i_ = 2.0090, a_N_ = 14.4 G, a_H_ = 2.22 G) appears due to the addition of the radical to PBN. The spectral parameters correspond to the N-CH_2_ radical [55]. During further irradiation, this signal gradually disappears, and the previously described multiplet appears, caused by a stable radical particle of the dye (Figure 6c). Irradiation of the AntTCNE/DMCHA/Iod in the presence of PBN yields similar spectra (Figure 6b,c). In this case, the triplet of doublets from the interception of free radicals by the trap has slightly different parameters (g_i_ = 2.0091, a_N_ = 14.4 G, a_H_ = 2.20 G), which corresponds to the phenyl radical. A similar signal is observed upon irradiation of a solution containing AntTCNE/Iod in the presence of PBN (Figure 6d).

**Scheme 3:**
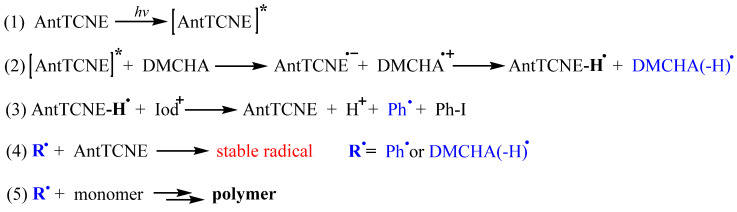
The proposed mechanism of photoinitiated polymerization of PETA in the presence of AntTCNEs.

Scheme 3 shows a proposed mechanism for photopolymerization in the presence of AntTCNE, based on an analysis of the above research results. In the first step, a photoexcited AntTCNE molecule reacts with an amine molecule to form tricyanoethyl and aminoalkyl radicals. The tricyanoethyl radical can be oxidized back to aryltricyanoethylene using an iodonium salt, resulting in the formation of a phenyl radical capable of initiating polymerization. The aminoalkyl radical can also initiate polymerization of the monomer. The phenyl radical, aminoalkyl and propagating radical can participate in the radical cyclization of tricyanoethylene, which results in the appearance of an EPR signal with three nonequivalent nitrogen atoms. It is worth noting that the possibility of reaction 3 Scheme 3 can explain the decrease in the observed rate of AntTCNE photodegradation upon the addition of Iod to the AntTCNE/DMCHA system while the photoinitiating efficiency of such a three-component system increases.

### 3.6. Two-Photon Polymerization

In general, all the studied ArTCNEs in combination with DMCHA and Iod initiate one-photon photopolymerization of PETA under irradiation in the 400 nm region. This is one of the conditions for initiating two-photon photopolymerization under the action of 780 nm titanium–sapphire laser radiation. We have previously shown that the PPCs with high reactivity in one-photon photopolymerization are also effective under conditions of two-photon photoinitiation of polymerization [56,57,58]. Accordingly, the most promising for research under two-photon photopolymerization are two- and three-component photoinitiating systems: AntTCNE/DMCHA, PhenTCNE/DMCHA, PyrTCNE/DMCHA, PerTCNE/DMCHA AntTCNE/DMCHA/Iod, and PyrTCNE/DMCHA/Iod.

Photopolymerization of the PPCs under two-photon photoinitiation conditions was studied using a Nanoscribe Photonics Professional. Using a 63× objective with a numerical aperture of NA = 1.4, the lithography modes for PPC were selected. The formulation of the PPC is presented in Table 5. The following parameters characterize the efficiency of recording structures during two-photon photopolymerization: The lower polymerization threshold is the minimum laser radiation power at which a polymer structure forms at a given beam scanning speed. Upper polymerization threshold (overexposure) is the laser radiation power at which the PPC boils. “Fabrication window” is the difference between the upper and lower polymerization thresholds. The dynamic range is the ratio of the “fabrication window” to the upper polymerization threshold, characterizing the portion of the power range suitable for nanolithography. To determine the thresholds for photopolymerization and overexposure, a test was performed in which both the laser power and the linear speed of lithography (a value inversely proportional to the exposure time) were varied. The fabrication windows at a scanning speed of 100 μm/s, as well as the formulation and designation of the studied PPCs, are presented in Table 5. When comparing two- and three-component photoinitiating systems **R1** and **R2** (AntTCNE), as well as **R4** and **R5** (PyrTCNE), it is clear that the introduction of iodonium salt into the PPC in the case of AntTCNE leads to an increase in the upper polymerization threshold, while for PyrTCNE the size of the processing windows does not change. For **R3** (PhenTCNE), the polymerization threshold could not be detected in the experiment: during the printing process, the polymer structures were formed only at radiation power in the overexposure range and they were not detected under development. The lowest polymerization threshold of 2 mW was for **R6** (PerTCNE); moreover, this PPC had the highest dynamic range.

The observed decrease in the lower polymerization threshold of PyrTCNE-based composites in the **R4** > **R1** > **R6** series toward AntTCNE and PerTCNE can be explained not only by the reactivity of these compounds under one-photon polymerization conditions (Table 4) but also by changes in the efficiency of two-photon absorption. The effective simultaneous absorption of two photons by an initiator molecule depends on the two-photon absorption cross-section: the higher the cross-section, the lower the two-photon polymerization threshold. The two-photon absorption cross-section is determined, among other things, by the molar extinction coefficient of the initiator at λ = λ_ext_/2. In the series of aryltricyanethylenes, the molar extinction coefficients at 390 nm increase to 2300 (PyrTCNE), 3350 (AntTCNE), and 5670 (PerTCNE) M^−1^cm^−1^. Accordingly, in the same series, the value of the lower polymerization threshold decreases.

**Figure 7:**
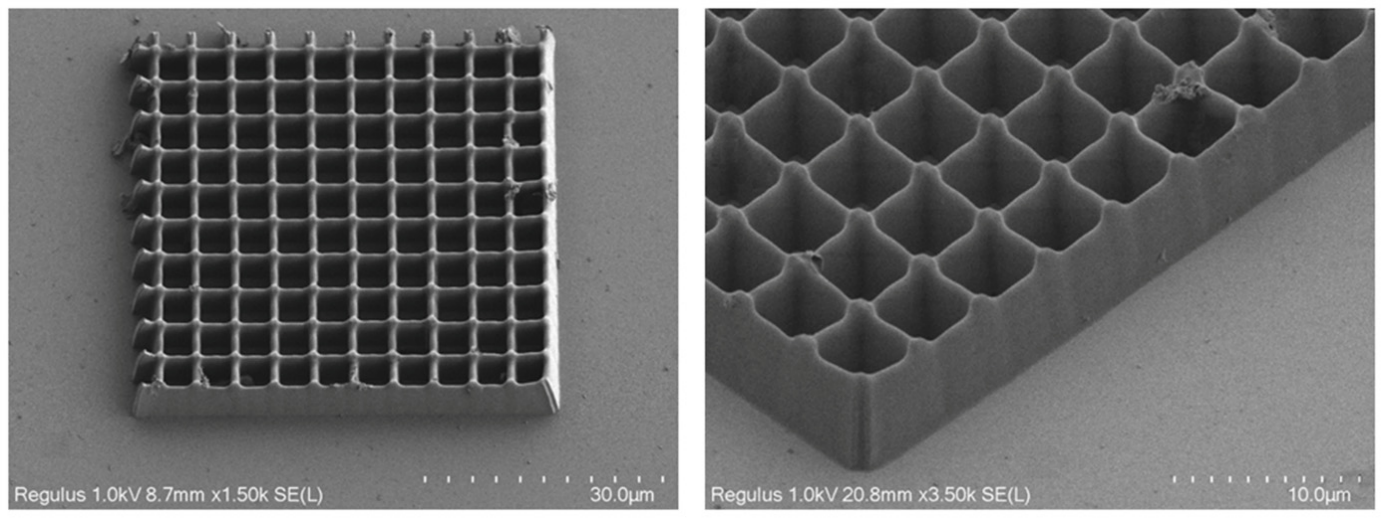
Fabricated test 3D structures from the **R2** at a magnification of ×1500 (**left**) and ×3500 (**right**).

Figure 7 shows images of the fabricated cage-type structures obtained with an electron microscope using **R2**. Structure parameters: cell period of 5 μm, overall size of 50 μm × 50 μm, and height of 7 ± 1 μm. Lithography parameters: 10 mW power and 100 µm/s scanning speed. The structures are stable and meet the parameters specified during manufacturing, which indicates the high potential for using this photocomposition for the production of 3D structures. The **R1** showed similar results.

**Figure 8:**
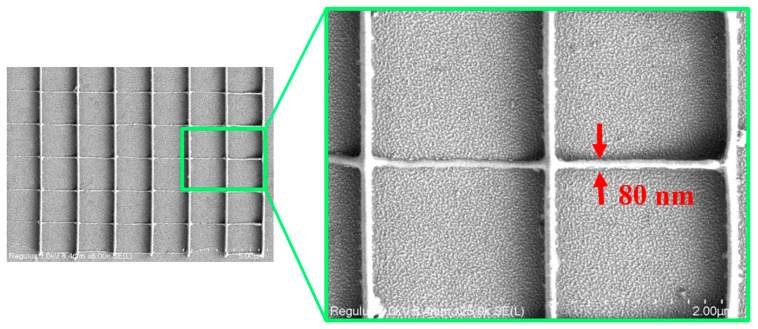
“Rail-sleeper” structures produced using the subthreshold drawing method from the **R1**.

Test “rail-sleeper” structures were also fabricated using the subthreshold drawing method to determine minimum feature sizes (subthreshold power of 8 mW, scanning speed of approximately 100 μm/s). Lines with a minimum width of 80 nm were obtained using **R1** (Figure 8).

**Figure 9:**
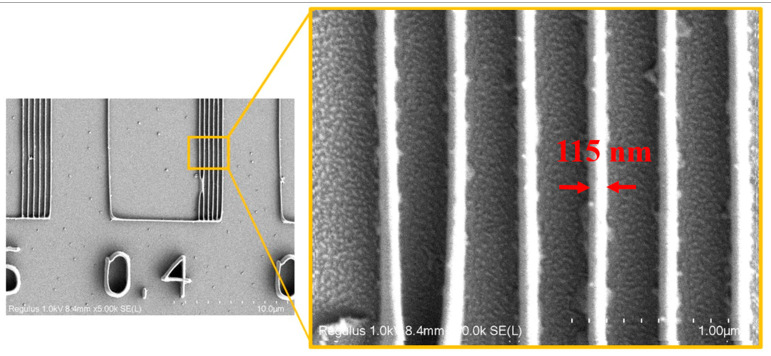
Fabricated structures for studying the line thickness in the **R1**.

To determine the lithography resolution and line thickness at different initiating radiation powers, polymer test structures were recorded (Figure 9). For the **R1** at a power of 9 mW and a scanning speed of 100 μm/s, the line thickness was 115 ± 10 nm with a distance between lines of 400 nm.

### 3.7. Cellular Cytotoxicity

**Figure 10:**
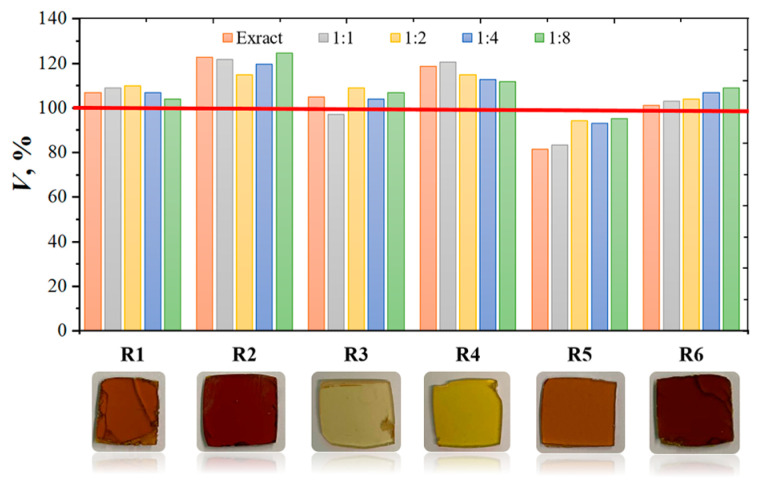
Viability of test culture cells after interaction with extracts of samples **R1**–**R6** and their dilutions.

For polymeric samples obtained from compositions **R1**–**R6**, the level of cytotoxicity was assessed. During the study, microscopic monitoring of the test culture’s condition was performed after exposure to extracts of **R1**–**R6** and their dilutions, which showed a comparable visual picture of the culture in the control and experimental series. The culture was a subconfluent monolayer of morphologically uniform cells, predominantly spindle-shaped, with prominent processes, dense nuclei, and homogeneous cytoplasms. No cell death or formation of large amounts of cellular debris was observed in any of the series. The colorimetric analysis results for the MTT assay were consistent with the microscopic data. The relative growth rate in all experimental series was ≥70%, meaning all samples were non-cytotoxic (Figure 10, Table 6). At the same time, a slight tendency toward stimulation of test culture cell proliferation upon interaction with extracts of samples **R2** and **R4** was observed, which persisted even upon dilution (Figure 10, Table 6). This fact deserves further investigation in subsequent work with these compounds in biomedical research.

## 4. Conclusions

In this study, the photoinitiating activity of aryltricyanoethylenes under one- and two-photon photopolymerization of PETA was comprehensively assessed for the first time. It was found that in the presence of DMCHA and/or Iod, all the studied dyes in acetonitrile solution photodegraded upon irradiation with LED@395 nm. Moreover, under one-photon photopolymerization conditions, the process occurred with the highest rate and maximum conversion using the three-component photoinitiating system ArTCNE/DMCHA/Iod compared to the two-component ArTCNE/DMCHA. This occurred due to the formation of additional phenyl radicals that initiated polymerization as a result of the interaction of dyes with the iodonium salt. Despite the “compactness” of the molecules, the studied aryltricyanethylenes also initiated two-photon photopolymerization. Moreover, PerTCNE exhibited the maximum reactivity and, accordingly, the minimum polymerization threshold, having the lowest oxidation potential of all ArTCNEs. A special place among the studied aryltricyanethylenes was occupied by AntTCNE, which was the least active in the photodegradation reaction and the most effective as a photoinitiator of single-photon photopolymerization. Despite the relatively low efficiency of AntTCNE under two-photon photopolymerization, PPCs with this dye were highly stable, which made it possible to obtain high-quality 3D nanostructures. Cellular scaffold structures were obtained by DLW nanolithography using AntTCNE-based PPC. For the same composition, structures with a minimum line width and line spacing of 80 and 400 nm, respectively, were recorded. According to the MTT test, the obtained polymeric materials showed promising preliminary biocompatibility and may warrant further biomedical evaluation. The knowledge gained from this study will serve as a basis for the development of new photoinitiators—tricyanoethylene anthracene derivatives—in our future work.

## Figures and Tables

**Table 1:** Maxima of the absorption bands of arylenes (Ar), maxima of the absorption bands of charge transfer (CT) in ArTCNEs and maxima of the fluorescence bands of ArTCNEs in acetonitrile, [ArTCNE] = 10^−5^ M. The fluorescence excitation wavelength is 400 nm.

Dye	λ_max_ (Ar), nm	λ_max_ (CT), nm (ε, M^−1^cm^−1^)	λ_max_ (Fluor), nm
**NaphTCNE**	270	358 (8160)	585
**AntTCNE**	375	525 (1900)	427
**PhenTCNE**	250	425 (6920)	485
**PyrTCNE**	~340, ~400	500 (5890)	460
**PerTCNE**	410	570 (6170)	500

**Table 2:** Redox potentials depending on Fc/Fc+ in acetonitrile (10^−3^ M) and the molecular orbital energies of ArTCNE calculated on their basis.

Dyes	E^1^_1/2 Red_, V	E_Ox_, V	E_HOMO_, eV	E_LUMO_, eV	ΔE, eV
**NaphTCNE**	−0.80	1.48	−6.58 **	−4.00	2.58
**PhenTCNE**	−0.83	1.46	−6.56 **	−3.97	2.59
AntTCNE *	−0.84	1.17	−5.97	−3.96	2.01
PyrTCNE *	−0.82	1.12	−5.92	−3.98	1.94
PerTCNE *	−0.80	0.82	−5.62	−4.00	1.62

*—values are taken from [46]. **—the value of the oxidation onset potentials (E_onset_). E_HOMO_ = −(E_[onset,ox vs_. _Fc+/Fc]_ + 5.1) (eV) [54].

**Table 3:** Effective initial photoconversion rate (*V*_0_) and half-life of ArTCNE (*τ*_1/2_) in the presence of amine (ArTCNE/DMCHA) and amine with iodonium salt (numerical values are given in brackets) under irradiation with LED@395 nm in acetonitrile. [ArTCNE] = 10^−4^ M; ArTCNE:DMCHA: Iod = 1:10:1 mol; P = 33 mW/cm^2^.

ArTCNE/DMCHA	*V*_0_ × 10^3^, c^−1^	*τ*_1/2_, c
**NaphTCNE** (+Iod)	6.6 (18.1)	65 (25)
**AntTCNE** (+Iod)	0.8 (0.12)	510 (830)
**PhenTCNE** (+Iod)	7.7 (11.4)	55 (35)
**PyrTCNE** (+Iod)	2.5 (8.9)	140 (50)
**PerTCNE** (+Iod)	6.8 (11.3)	60 (40)

**Table 4:** Kinetic parameters of photopolymerization of PETA in the presence of the initiating systems ArTCNE/DMCHA, ArTCNE/Iod and ArTCNE/DMCHA/Iod. The light source is at 405 nm with 33 mW/cm^2^ irradiation intensity. [ArTCNE] = 0.018 M; [Am] = 0.18 M; [Iod] = 0.018 M.

	W_max_ × 10^2^, s^−1^	P (15 min), %
**ArTCNE/DMCHA**		
NaphTCNE/DMCHA	14.6 ± 0.7	25 ± 1
AntTCNE/DMCHA	97.1 ± 4.4	48 ± 2
PhenTCNE/DMCHA	75.4 ± 3.7	40 ± 2
PyrTCNE/DMCHA	80.0 ± 3.6	50 ± 2
PerTCNE/DMCHA	79.3 ± 3.9	39 ± 1
**ArTCNE/Iod**		
NaphTCNE/Iod	18.7 ± 0.7	26 ± 1
AntTCNE/Iod	18.5 ± 0.6	33 ± 1
PhenTCNE/Iod	18.2 ± 0.7	24 ± 1
PyrTCNE/Iod	5.9 ± 0.3	7 ± 1
PerTCNE/Iod	19.3 ± 0.9	26 ± 1
**ArTCNE/DMCHA/Iod**		
NaphTCNE/DMCHA/Iod	121.7 ± 5.9	40 ± 2
AntTCNE/DMCHA/Iod	120.0 ± 5.4	46 ± 2
PhenTCNE/DMCHA/Iod	125.0 ± 5.6	43 ± 2
PyrTCNE/DMCHA/Iod	120.0 ± 5.3	52 ± 2
PerTCNE/DMCHA/Iod	123.0 ± 5.5	45 ± 2

**Table 5:** Fabrication windows (FW) for **R1**–**R6** at a laser beam scanning speed of 100 µm/s.

No.	Formulation of PPCs	FW, mW	Dynamic Range
**R1**	**AntTCNE**/DMCHA/PETA	8 ÷ 13.5	0.40
**R2**	**AntTCNE**/DMCHA/Iod/PETA	7.5 ÷ 17.5	0.57
**R3**	**PhenTCNE**/DMCHA/PETA	20 ÷ 20	-
**R4**	**PyrTCNE**/DMCHA/PETA	9 ÷ 16	0.43
**R5**	**PyrTCNE**/DMCHA/Iod/PETA	9 ÷ 16	0.43
**R6**	**PerTCNE**/DMCHA/PETA	2 ÷ 13	0.84

**Table 6:** Evaluation of the cytotoxicity of powdered samples **R1**–**R6** during 1-day extraction using the MTT test.

Sample	Parameter	R1	R2	R3	R4	R5	R6
**Control (n = 8)**	**OD**	0.786 ± 0.016	0.683 ± 0.012	0.780 ± 0.020	0.653 ± 0.014	0.855 ± 0.021	0.783 ± 0.013
	***V,*** **%**	100	100	100	100	100	100
	**Rank of cytotoxicity**	0	0	0	0	0	0
**Extract (n = 8)**	**OD**	0.858 ± 0.014	0.855 ± 0.017	0.852 ± 0.011	0.793 ± 0.021	0.711 ± 0.013	0.806 ± 0.010
	***V,*** **%**	109	125	107	121	83	103
	**Rank of cytotoxicity**	0	0	0	0	1	0
**Extract 1:1** **(n = 8)**	**OD**	0.872 ± 0.022	0.849 ± 0.009	0.789 ± 0.007	0.804 ± 0.018	0.728 ± 0.010	0.822 ± 0.060
	***V,*** **%**	111	124	99	123	85	105
	**Rank of cytotoxicity**	0	0	1	0	1	0
**Extract 1:2** **(n = 8)**	**OD**	0.879 ± 0.032	0.799 ± 0.006	0.884 ± 0.012	0.761 ± 0.006	0.820 ± 0.011	0.829 ± 0.011
	***V,*** **%**	112	117	111	117	96	106
	**Rank of cytotoxicity**	0	0	0	0	1	0
**Extract 1:4** **(n = 8)**	**OD**	0.858 ± 0.025	0.832 ± 0.016	0.850 ± 0.008	0.752 ± 0.009	0.808 ± 0.007	0.857 ± 0.010
	***V,*** **%**	109	122	106	115	95	109
	**Rank of cytotoxicity**	0	0	0	0	1	0
**Extract 1:8** **(n = 8)**	**OD**	0.834 ± 0.021	0.870 ± 0.041	0.875 ± 0.006	0.749 ± 0.010	0.831 ± 0.010	0.867 ± 0.011
	***V,*** **%**	106	127	109	114	97	111
	**Rank of cytotoxicity**	0	0	0	0	1	0

## Data Availability

Data are contained within the article and Supplementary Materials.

## Supplementary Materials

The following supporting information can be downloaded at https://www.mdpi.com/article/10.3390/polym18080958/s1: Figure S1: DFT calculations for PhenTCNE. Figure S2: The spectral changes in the AntTCNE (a) and AntTCNE/Iod (b) solutions in acetonitrile under irradiation with LED@395 nm. Figure S3: The change in intensity of the absorption band of acrylate groups at 806 cm−1 relative to the unchanged intensity of the band corresponding to vibrations of the C=O group at 1720 cm−1 during photopolymerization of PETA initiated by ArTCNE/DMCHA.
