# Supplementary material for: Aromatic Tricyanoethylenes a New Class of ‘Compact’ Photoinitiators for One- and Two-Photon Photopolymerization
Source: Polymers (Basel). 2026 Apr 14;18(8):958. doi: 10.3390/polym18080958 (PMC13119627; doi:10.3390/polym18080958)
Supplement: Supplementary file 1 [file polymers-18-00958-s001.zip › polymers-4213243-supplementary.pdf]

Supplementary Material

Aromatic Tricyanoethylenes a New Class of ‘Compact’ Photoinitiators for One- and Two-Photon Photopolymerization

Elnara R. Zhiganshina [1], Tatyana S. Lyubova [1], Anastasia E. Tarakanova [1], Maxim V. Arsenyev [1,*], Roman S. Kovylin [1], Natalia D. Anisimova [1], Yuri V. Polushtaytsev [1], Konstantin A. Kozhanov [1], Anastasia V. Pisarenko [2], Diana Ya. Aleynik [3], Marfa N. Egorikhina [3], Alexei Vitukhnovsky [4], Larisa G. Klapshina [1] and Sergey A. Chesnokov [1]

1 G.A. Razuvaev Institute of Organometallic Chemistry, Russian Academy of Science (RAS), Tropinin St., 49, 603137 Nizhny Novgorod, Russia; zhiganshinae@mail.ru (E.R.Z.); lyubovat@rambler.ru (T.S.L.); anasta5ia.tar@yandex.ru (A.E.T.); mulnir@yandex.ru (R.S.K.); and@iomc.ras.ru (N.D.A.);
jurik_sib@mail.ru (Y.V.P.); kostik@iomc.ras.ru (K.A.K.); klarisa@iomc.ras.ru (L.G.K.);
sch@iomc.ras.ru (S.A.C.)

2 Moscow Institute of Physics and Technology, National Research University, Institutskii Per. 9,
141700 Dolgoprudny, Russia; pisarenko.nastya@gmail.com

3 Privolzhsky Research Medical University of the Ministry of Health Care of the Russian Federation, Minin and Pozharsky Sq. 10/1, 603950 Nizhny Novgorod, Russia; daleynik@yandex.ru (D.Y.A.);
egorihina.marfa@yandex.ru (M.N.E.)

4 Lebedev Physical Institute, Russian Academy of Sciences, Leninskii Prospect 53, 119991 Moscow, Russia; vitukhnovsky@mail.ru

* Correspondence: mars@iomc.ras.ru

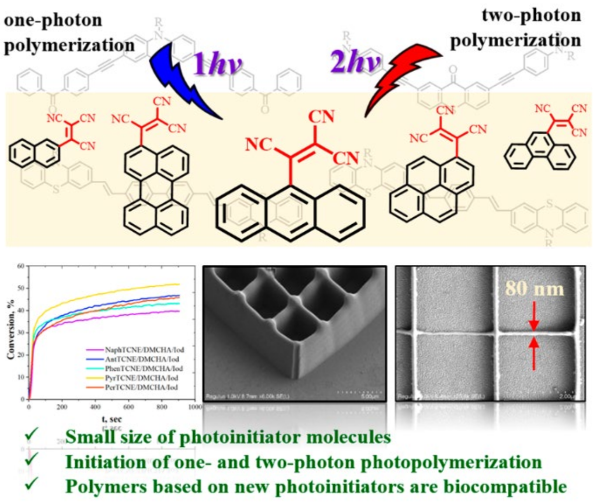

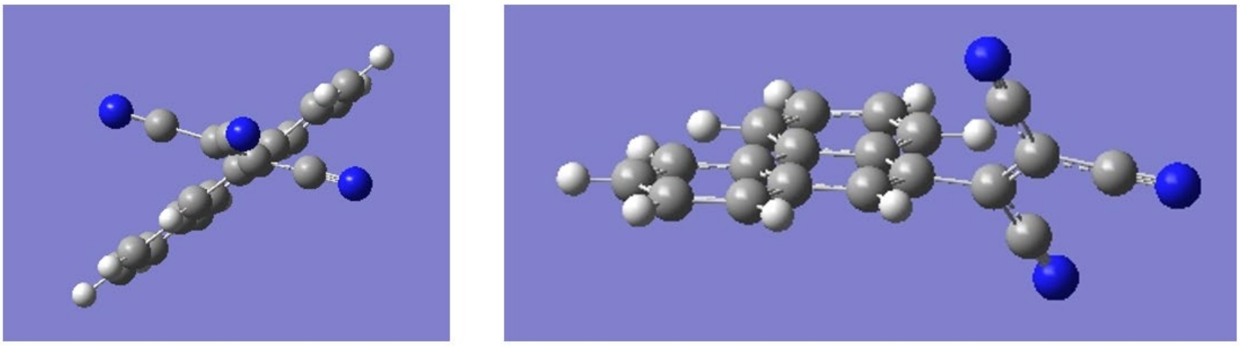

Figure S1. DFT calculations were performed using the Gaussian 09 software package with the B3LYP hybrid functional and the 6-31G (d,p) basis set for all atoms. The absence of imaginary frequencies indicates that the molecules are at minimum potential energy. The dihedral angle between the planes of the tricyanoethylene and the plane of phenanthrene is **52.5(5)°.**

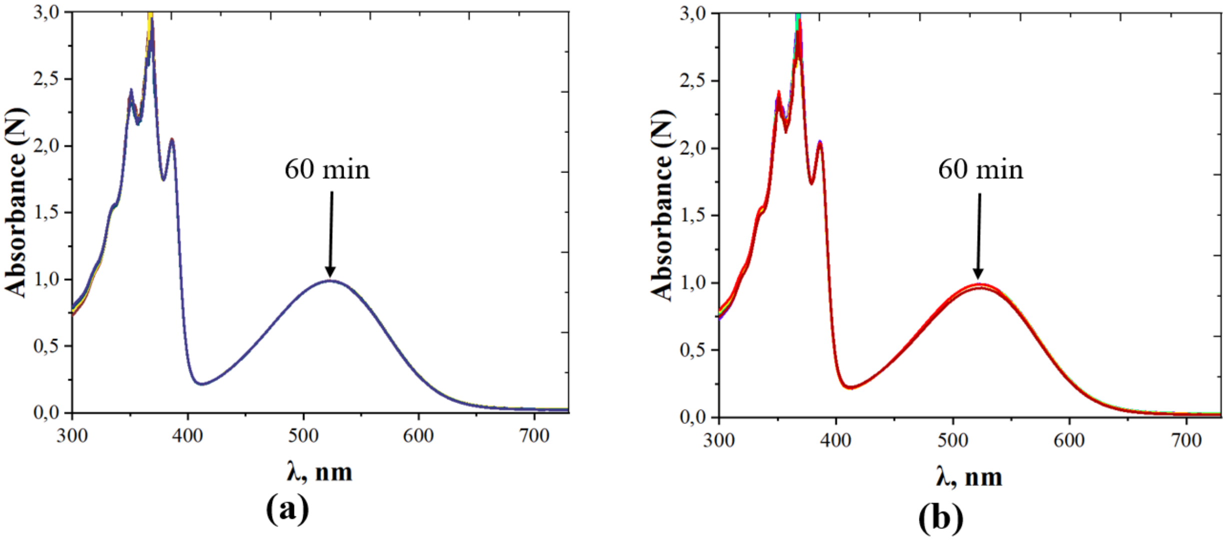

Figure S2. The spectral changes of the AntTCNE (a), AntTCNE/Iod (b) solutions in acetonitrile under irradiation with LED@395 nm. ArTCNE : Iod = 1:1 mol; P = 33 mW/cm^2.

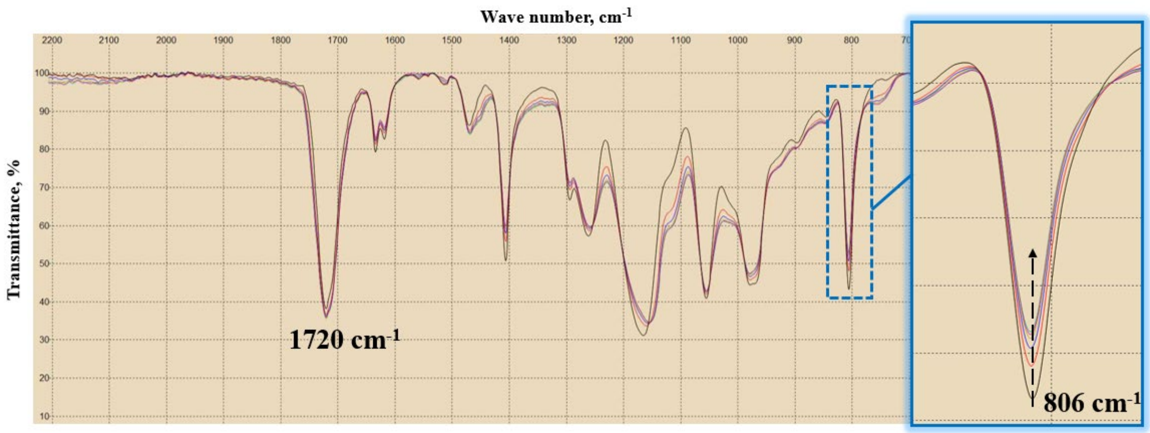

Figure S3. The change in intensity of the absorption band of acrylate groups at 806 cm^{-1} relative to the unchanged intensity of the band corresponding to vibrations of the C=O group at 1720 cm^{-1} during photopolymerization of PETA initiated by **ArTCNE/DMCHA**.
